# Spontaneous inflammatory pain model from a mouse line with *N*-ethyl-*N*-nitrosourea mutagenesis

**DOI:** 10.1186/1423-0127-19-55

**Published:** 2012-05-30

**Authors:** Tsung-Chieh Chen, José Jiun-Shian Wu, Wei-Pang Chang, Ping-Ning Hsu, Sung-Tsang Hsieh, Bai-Chuang Shyu

**Affiliations:** 1 Institute of Biomedical Sciences, Academia Sinica, Taipei, 11529, Taiwan, Republic of China; 2 Institute of Zoology, National Taiwan University, Taipei, Taiwan, Republic of China; 3 Graduate Institute of Life Science, National Defense Medical Center, Taipei, Taiwan, Republic of China; 4 Department of Internal Medicine, National Taiwan University Hospital and Graduate Institute of Immunology, National Taiwan University, Taiwan, Republic of China; 5 Department of Neurology, National Taiwan University Hospital and Department of Anatomy and Cell Biology, National Taiwan University, Taiwan, Republic of China

**Keywords:** *N*-ethyl-*N*-nitrosourea, Autoinflammation, *pstpip2*, Single-nucleotide polymorphism, Nociception, Inflammatory pain

## Abstract

**Background:**

*N*-ethyl-*N*-nitrosourea mutagenesis was used to induce a point mutation in C57BL/6 J mice. Pain-related phenotype screening was performed in 915 G3 mice. We report the detection of a heritable recessive mutant in meiotic recombinant N1F1 mice that caused an abnormal pain sensitivity phenotype with spontaneous skin inflammation in the paws and ears.

**Methods:**

We investigated abnormal sensory processing, neuronal peptides, and behavioral responses after the induction of autoinflammatory disease. Single-nucleotide polymorphism (SNP) markers and polymerase chain reaction product sequencing were used to identify the mutation site.

**Results:**

All affected mice developed paw inflammation at 4–8 weeks. Histological examinations revealed hyperplasia of the epidermis in the inflamed paws and increased macrophage expression in the spleen and paw tissues. Mechanical and thermal nociceptive response thresholds were reduced in the affected mice. Locomotor activity was decreased in affected mice with inflamed hindpaws, and this reduction was attributable to the avoidance of contact of the affected paw with the floor. Motor strength and daily activity in the home cage in the affected mice did not show any significant changes. Although Fos immunoreactivity was normal in the dorsal horn of affected mice, calcitonin gene-related peptide immunoreactivity significantly increased in the deep layer of the dorsal horn. The number of microglia increased in the spinal cord, hippocampus, and cerebral cortex in affected mice, and the proliferation of microglia was maintained for a couple of months. Two hundred eighty-five SNP markers were used to reveal the affected gene locus, which was found on the distal part of chromosome 18. A point mutation was detected at A to G in exon 8 of the *pstpip2* gene, resulting in a conserved tyrosine residue at amino acid 180 replaced by cysteine (Y180 C).

**Conclusions:**

The data provide definitive evidence that a mutation in *pstpip2* causes autoinflammatory disease in an *N*-ethyl-*N*-nitrosourea mutagenesis mouse model. Thus, our *pstpip2* mutant mice provide a new model for investigating the potential mechanisms of inflammatory pain.

## Background

Pain and hyperalgesia frequently result from peripheral tissue injury or nerve damage [1,2]. These abnormal sensory events arise partially from the action of inflammatory mediators on peripheral terminals of nociceptive primary sensory neurons [3,4]. Numerous molecules, such as prostaglandins and bradykinin, are known to be produced by tissue damage or inflammation and considered responsible for the activation and sensitization of peripheral pain-signaling sensory neurons [5]. Newly identified mediators include various factors produced and released from non-neuronal cells, such as macrophages and immune cells. These factors can act at numerous loci and play important roles in mediating persistent pain states [6,7].

Chronic pain includes neuropathic pain, inflammation pain, and cancer pain and is often not adequately treated by currently available analgesics. Several mouse and rat models have been used in the study of the mechanisms of chronic pain [8]. Inflammatory pain can be induced in animals by complete Freund’s adjuvant (CFA) and carrageenan injection, which cause acute inflammatory pain and chronic polyarthritis [8,9]. Chronic inflammation may also lead to an enhanced A- and C-fiber response and behavioral hypersensitivity in the CFA injection animal model [10]. Glial cells are excited by pro-inflammatory cytokines and neuroactive factors, which have been shown to maintain and induce chronic pain [11,12]. Inflammatory diseases cause progressive damage to neighboring nerve tissues and organs in humans [13,14]. In classic neuroimmune diseases, such as multiple sclerosis and acute disseminated encephalomyelitis, neuroantigen-reactive lymphocytes escape immune tolerance, invade the central nervous system, and are responsible for tissue damage, demyelination, and axonal degeneration [15].

Recently, transgenic models have proven to be useful experimental methods in the study of pain. The knockout of specific nociception-related genes has been used to unravel the underlying mechanism of pain [16,17]. Treatment with *N*-ethyl-*N*-nitrosourea (ENU) efficiently generates single-nucleotide mutations in mice, which can then be screened for phenotypes of pain-related inflammatory disease. A systematic genome-wide and phenotype-driven mouse mutagenesis program for gene function has been described [18,19]. We participated in a large-scale ENU mutagenesis program sponsored by the National Science Council of Taiwan and Academia Sinica. The aim of the present study was to identify genes related to chronic inflammatory syndromes induced by ENU in mice. Different nociceptive assay screening procedures were used to compare wildtype and ENU mutant mice and select abnormal nociceptive function. Four different nociceptive assays were used to test thermal and mechanical nociceptive sensitivity in ENU-treated mice. The tail-flick and hot plate tests assess thermal pain. The von Frey and tail pressure assays assess mechano-sensitive nociception. We detected an ENU-treated mouse line with a spontaneous inflammatory syndrome, providing pathophysiological evidence in the organs and tissue of the mutant mice. These mice developed spontaneous inflammation in the extremities at approximately postnatal weeks 4–5. We further investigated behavioral responses and neuronal peptides after the induction of autoinflammatory disease. The mutant mice displayed a *Mayp/pstpip2* point mutation that resulted in autoinflammatory disease. These mice have been bred to the 10th generation, and a consistent phenotype has been maintained.

## Methods

### Mice

ENU-treated mice were bred according to the three-generation breeding scheme as described by Weber et al. (2000). Briefly, C57BL/6 J (B6) male mice were given three intraperitoneal ENU injections (100 mg/kg body weight) to generate G0 mice. G0 mice were then mated with normal B6 females to generate male G1 founder mice. Normal B6 females were mated with male G1 founders to generate G2 mice. G2 females were then backcrossed to male G1 mice to generate G3 offspring. Four different nociceptive assays were used to test thermal and mechanical nociceptive sensitivity in G3 mice. All of the testing data were standardized, and values greater or less than 2.5-times the standard deviation were defined as deviant and subjected to a further heritability test. The slow (or fast) response was defined as the response value of the deviant higher (or lower) than the mean. Mice with the phenotype selected from the screening protocol were mated with mice from the same genetic background and bred to produce G4 mice. The G5 generation was outcrossed with C3H/HeN to dilute the genetic background and produce F1 hybrid carrier mice. The F1 generation was then intercrossed with siblings to breed the affected N1F1 mice. The outcross strategy was used for single-nucleotide polymorphism (SNP) mapping. The breeding and housing of the mice occurred in the Mouse Mutagenesis Program Core facility of Academia Sinica under specific pathogen-free conditions. The mice were maintained on a regular rodent diet. All of the experiments were performed in accordance with the guidelines of the Academia Sinica Institutional Animal Care and Utilization Committee (PicoLab Mouse Diet 20 code 5058, PMI LabDiet, St. Louis, MO, USA).

### Inflammation measurement

The width of the first digit in affected and unaffected mice was measured from week 4 to week 20. Graph paper with a 1 mm scaled grid was used as the background for photographs of the digits (Nikon Coolpix 990). The width of the digit was calculated from digital images using ImageJ software (National Institutes of Health, Bethesda, MD, USA).

### Histology

Anesthetized animals were perfused with 0.9% NaCl and subsequently 4% paraformaldehyde in 0.1 M phosphate-buffered saline (PBS; pH 7.4), followed by decalcification using 20% ethylenediaminetetraacetic acid, which was frequently changed for 2 weeks. Histopathologic examinations were performed followed by hematoxylin/eosin (H&E) staining.

### X-ray analysis

To detect bone morphology in affected and unaffected mice, X-ray radiography was used to identify the hindpaws of the mice. The Radiography X-ray system (GTR Labs, KV = 38, MAS = 1.25) and Kodak Medical X-ray Film General Purpose Green (30.5 cm × 38 cm) were used to expose the image.

### Magnetic resonance imaging

All magnetic resonance imaging (MRI) experiments were performed using a 4.7 T Biospec 47/40 spectrometer (Bruker, Germany) with an active shielding gradient (20 G/cm, 80 μs rise time). Under isoflurane anesthesia (1.5-2.0% in oxygen), the body temperature (37°) and respiratory rate (40-55/min) of the mice were monitored and maintained throughout the experiment. Multi-slice T2-weighted images were obtained with a 2 cm field-of-view, 1 mm slice thickness, and 128 × 128 data matrix.

### Mechanical nociceptive behavioral assays

The von Frey assay was used to assess mechanical nociceptive sensitivity. A von Frey filament was attached to a force transducer (Model 1601 C, IITC, Woodland Hills, CA, USA). The hindpaw was poked with a von Frey filament until paw withdrawal was seen. The force that initiated paw withdrawal was considered the threshold value. The threshold of the von Frey test was determined as the mean value from 10 trials.

### Tail-flick test

The tail-flick test was designed to assess thermal nociceptive sensitivity using a tail-flick apparatus (Model 37360, Tail Flick Unit, Ugo Basile, Italy). The mice were allowed 15 min to acclimatize to the apparatus. The heat intensity was set to 15 units, and the cut-off time was set to 22 s. The latency was recorded when the tail was flicked away from the heat source.

### Hot plate test

This test was used to measure thermal nociception. A commercially available hot/cold plate apparatus was used (35100 Hot/Cold Plate, Ugo Basile, Italy). The hot plate apparatus was switch on to heat the surface of the hot plate to a constant temperature of 55 ± 0.2°C. The mice were placed on the hot plate (20 cm diameter), which was surrounded by a clear acrylic protective casing (25 cm height with an open top). The paw withdrawal latency, reflected by hindpaw licking, flicking, or jumping, was recorded.

### CatWalk analysis

The gait of auto-inflammatory pain and wildtype mice was detected using CatWalk methods (Noldus Information Technology, Leesburg, VA, USA). The mice were placed in a chamber with a glass floor, which have light source alone the glass edge. The light could be reflected internally to downward directions during the paw or tail contact with the glass surface. The more reflected intensity and more brighter pixels were revealed, the more press is in the contact region. The movements of the test mice were recorded by a video camera under the glass floor. Paw contact with the floor was calculated using ImageJ software.

### Open field test

The mice were placed in Plexiglas chambers (48 cm × 48 cm) that had a 16 × 16 array of horizontal sensors. Vertical and horizontal motor activity was calculated as the total number of beams breaks in the X-Y plane in 10 ms bins [20].

### Rotarod test

A commercially available Rota Rod apparatus (47600 Rota-Rod, Ugo Basile, Italy) was used. The diameter of the rotating rod was approximately 5 cm, which was made of hard plastic material covered by gray rubber foam. The lane width was 5 cm. The apparatus allowed an accelerating speed of 4 rotations per minute (rpm) to 40 rpm in 300 s. The time to fall from the rotarod was recorded.

### Homecage scan

The homecage scanning system monitors daily behaviors in mice when placed in a safe and comfortable environment (i.e., 12 h/12 h light/dark cycle with constant temperature and humidity). Clever Sys HomeCageScan TM3.0 software (Clever Sys., Inc., Reston, VA, USA) was used for monitoring. Six behaviors, including feeding, drinking, walking, rearing, hanging, and grooming, were detected simultaneously by this system. The ratio of the time engaged in each activity during the light and dark periods was calculated and compared.

### Immunohistochemistry

To detect pathological changes in the affected mice, rat anti-F4/80 antibody (eBioscience, San Diego, CA, USA), rabbit anti-Iba1 antibody (Abbiotec, San Diego, CA, USA), rabbit anti-CGRP antibody (Chemicon International, Temecula, CA, USA), and rabbit anti-Fos antibody (Santa Cruz Biotechnology, Santa Cruz, CA, USA) were used. Affected homozygous, control unaffected homozygous, and heterozygous animals were anesthetized and perfused with 0.9% NaCl and subsequently 4% paraformaldehyde in 0.1 M PBS (pH 7.4). The skin, paws, and organs were removed, postfixed for 72 h, sectioned at 10 μm on a cryostat (Leica CM3050S, Leica Microsystems, Nussloch, Germany), and processed for immunohistochemistry. The sections were incubated with 0.1% trypsin for 20 min at 37 °C. The sections were then cooled to room temperature for 20 min. Normal serum was added for 1 h, and the solution was reacted with rat anti-F4/80 antibody overnight at 4 °C. Biotinylated immunoglobulin G (IgG) was used for 1 h at room temperature. Immunohistochemistry was performed using avidin-biotin elite solution for 1 h (ABC kit, Vector Laboratories, Burlingame, CA, USA). The stainings were visualized using 0.03% 3,3-diaminobenzidine (DAB) and 0.07% H_2_O_2_ in Tris buffer (pH 7.4). The distribution and intensity of immunoreactivities were analyzed using ImageJ software.

### SNP genotyping

Genomic DNA was purified from the tails using the Puregene DNA purification kit (Gentra Systems, Minneapolis, MN, USA). Two hundred eighty-five genome-wide mouse SNP markers were used to identify the mutation site, and we selected nine SNP markers located in the distal part of chromosome 18 for the fine mapping of SNP genotypes. All of the SNPs were genotyped using a MassARRAY (Sequenom, San Diego, CA, USA).

### Sequencing of polymerase chain reaction products

Genomic DNA was purified from the tails using the Puregene DNA purification kit (Qiagen [Gentra Systems], Minneapolis, MN, USA). All PCR reactions were performed with recombinant Taq DNA polymerase (MBI Fermentas) for 35 cycles. The exons of candidate genes (*pstpip2*) were amplified, and the primers of candidate genes were designed with primer3 software. All PCR primers were synthesized by Research Biolabs (Singapore). Using 2% ethidium bromide-stained agarose gel electrophoresis, a 123–400 base pair (bp) PCR fragment length of the affected sequence region was produced and excised for gel extraction. Genomic DNA was extracted from the cutting gel using a DNA extraction kit (Genemark). The extracted PCR product was diluted to 1–3 ng/100 bp and contained 2 ng/μl as the template for the sequence reaction (ABI PRISM 3700 DNA Analyzer, Applied Biosystems, Foster City, CA, USA).

### Data analysis

ImageJ image analysis software was used to quantify the intensity of immunoreactivity in the regions of interest. To prevent immunostaining variations in different samples, the values of the immunoreactivity intensity were normalized by calculating the ratio of the intensity in reactive tissues and inactive tissues (e.g., white matter). Five standard unit areas (a 200 μm × 200 μm square) were randomly assigned and placed on the region of interest so that these five unit areas were covered as evenly as possible on the region of interest. The immunoreactivities measured from these five unit areas were averaged to obtain the mean immunoreactivity value in specific regions of interest. Quantitative data were analyzed using unpaired *t*-tests. All of the data are expressed as the mean ± SEM, and significant differences were determined using Student’s *t*-test. Values of *p* < 0.05 were considered statistically significant. One-way analysis of variance (ANOVA) was used to analyze the incidence of autoinflammation and onset of autoinflammation. When appropriate, Tukey’s *post hoc* test was used.

## Results

### Mutant phenotype

A total of 915 G3 mice (463 male and 452 female) were used to screen abnormal nociceptive responses. Eight of the mice exhibited a fast nociceptive response. These mice with abnormal nociceptive responses were selected to generate G4 mice. The G4 mice were mated with G3 fathers and mothers as the backcross strategy to produce G5 deviants. In the G5 generation, we selected the mice with a hypersensitive nociceptive response for the purpose of SNP mapping. These mutant mice were then outcrossed with C3H/HeN mice to produce the N1 generation. Intercross breeding was conducted to breed the affected homozygous mice. In the N1F1 generation (*n* = 62), eight female and two male mutant mice showed an abnormal phenotype (i.e., spontaneous skin inflammation in the four paws and ears). The offspring of N2 mice (i.e., the affected N1F1 mice outcrossed with C3H/HeN mice) did not display a mutant phenotype. The percentage of affected mice (20.6%, 18 affected mice among 87 N2F1 offspring) in the N2F1 generation indicated that the autoinflammatory syndrome was a recessive mutation.

**Figure 1:**
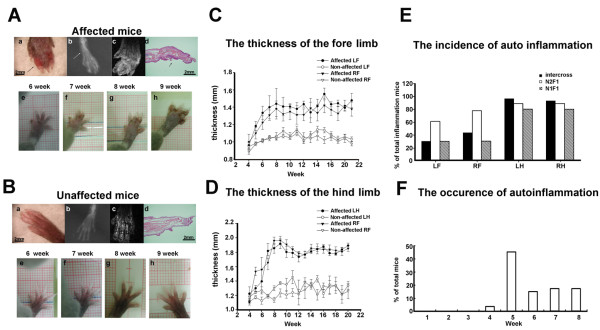
**Phenotype of paw morphology in unaffected and affected mice.** Mouse detected by X-ray (**A,*b***), MRI (**A,*c***), and H&E staining (**A,*d***). (**A**) (***a***) Clinical symptoms of edematous inflammation and necrosis of the digits (arrows). (***b***) Radiography of a paw in an affected mouse shows bone destruction, and the shadow shows enlarged soft tissue. (***c***) Affected mice exhibited indistinct signals of swollen soft tissue revealed by MRI. (***d***) Inflamed phalanges of H&E stains in affected mice. The arrows show hyperplasia of the epithelium. (**A,*e-h***) Paw development in unaffected offspring from week 6 to week 9. (**B,*a-h***) Comparison of respective phenotypes in unaffected mice. (**C**) Thickness of the digits in the inflamed forelimb in mice with autoinflammation and unaffected mice. (**D**) Thickness of the digits in the inflamed hindlimb in affected and unaffected mice. (**E**) Percentage of penetrance in each limb in affected mice. (**F**) Age of onset of clinical symptoms in affected mice. Abbreviations: LF, left forelimb; RF, right forelimb; LH, left hindlimb; RH, right hindlimb.

The affected homozygous mutant mice all showed spontaneous inflammatory signs, including red, swollen, and deformed paws (Figure 1A, *a*). X-ray analysis revealed bone destruction and shadows that indicated enlarged soft tissue in affected mice (Figure 1A, *b*). Using MRI, the inflammation area of the soft tissue was abnormal compared with the paws of unaffected mice (Figure 1A, *c*). H&E staining showed proliferation in the epithelial layer of the paw, and abnormal, deeply dense cells were found in the inflamed area (Figure 1A, *d*). Figure 1B, *e-h*, shows the condition of the paw in an affected mouse from week 6 to week 9. The phenotypes of the unaffected mice that were littermates of affected mice and heterozygous for the mutation are shown in Figure 1B. Six affected mice and four unaffected mice were used in the X-ray analysis. Two affected mice and two unaffected mice were evaluated by MRI. Seven affected mice and four unaffected mice were used in the H&E staining study. The mutant mice developed paw deformities that began in week 5 to week 8. Subsequently, the mice developed thickened, dystropic hindpaws. The mutant paws displayed inflammation symptoms in the digits that also affected the widths of the digits. Figure 1C shows the thickness of the digits in the inflamed forelimbs in affected mice (*n* = 20) and unaffected mice (*n* = 8). Figure 1D shows the thickness of the digits in the inflamed hindlimbs in affected mice (*n* = 20) and unaffected mice (*n* = 8). The data analysis showed that the affected mice had significantly thicker paws than wildtype mice. The long-term development of the thickness of the paws in the fore- and hindlimbs in the affected mice also showed that this syndrome was irreversible. The percentage of penetrance in each limb of the affected mice showed that most of the affected mice had autoinflammation symptoms in the hindlimbs (Figure 1E; intercross mice, *n* = 30; N1F1 mice, *n* =18; N2F1 mice, *n* = 10). Figure 1E shows the mean ± SEM percentage of the incidence of total autoinflammation in the four limbs in intercross, N2F1, and N1F1 mice. One-way ANOVA indicated a significant difference (*F*_3,116_ = 27.71, *p* < 0.05) in intercross mice. Furthermore, the *post hoc* tests revealed a significant difference between the forelimbs and hindlimbs (*p* < 0.05). In N1F1 mice, a one-way ANOVA revealed significant differences between the left forelimb, right forelimb, left hindlimb, and right hindlimb (*F*_3,39_ = 4.05, *p* < 0.05). However, in N2F1 mice, one-way ANOVA revealed no significant differences between the left forelimb, right forelimb, left hindlimb, and right hindlimb (*F*_3,71_ = 1.93, *p* > 0.05). The phenotype of the mutant mice could be identified as early as postnatal week 4, and the first symptoms were observed during week 4 to week 8 (Figure 1F; *n* = 51). Inflammation in the affected mice mostly occurred in week 5.

### Pathology of autoinflammation

**Figure 2:**
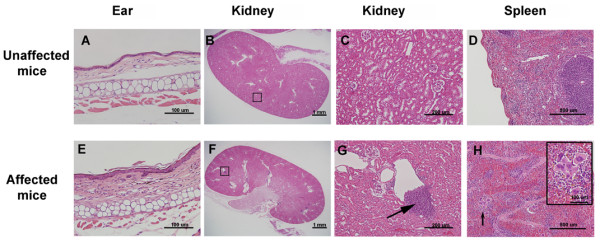
**Pathology data of organ tissues in unaffected and affected mice.** (**A and E**) Mice with autoinflammation showed proliferation in the epithelial layer of the ear. (**B, C, F, and G**) Cell accumulations were detected in the kidney (square in F and arrow in enlarged picture in **G**). (**D and H**) H&E staining demonstrated that autoinflammatory disease caused megakaryocyte proliferation in the red pulp of the spleen (arrow; **H** and inset).

Histologic analysis revealed that cell proliferation occurred in the epithelial layer of the ears in affected mice (Figure 2E) compared with unaffected mice (Figure 2A). Cell accumulation was detected in the kidneys of affected mice (Figure 2F and G, arrow) compared with unaffected mice (Figure 2B and C). In the spleen, more megakaryocytes appeared in the red pulp of affected mice (Figure 2H, arrow and inset) compared with unaffected mice (Figure 2D). This megakaryocyte proliferation phenomenon was also reported in chronic multifocal osteomyelitis (*cmo*) mice [21]. The data from unaffected mice are shown in Figure 2A-D. Seven affected mice and four unaffected mice were used for H&E staining. Thus, autoinflammatory disease appeared to cause megakaryocyte proliferation in the red pulp of the spleen in H&E-stained sections. Other histology data, including in the skin, thymus, lungs, pancreas, liver, kidneys, heart, and joints, were normal in affected mice.

### Immunostaining of macrophage marker F4/80

**Figure 3:**
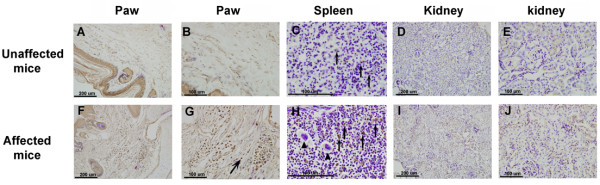
**F4/80 immunostaining and hematoxylin counterstaining of organ tissues of affected and unaffected mice.** (**A-E**) The upper panel shows immunoreactivity in unaffected mice. (**F-J**) The lower panel shows the immunoreactivity of mice with autoinflammatory disease. In the paw section, the affected mice showed increased immunoreactivity (**F** and **G**, arrow). F4/80 immunoreactivity also increased in the spleen in affected mice (**H**, arrow). Megakaryocytes were also detected in affected mice (**H**, arrowhead). In the kidney section, F4/80 immunoreactivity was unchanged in unaffected mice (**D** and **E**) and mice with autoinflammation (**I** and **J**).

We then sought to determine whether macrophages are related to the observed inflammatory syndromes. Immunostaining of the F4/80 antigen and counterstaining with hematoxylin were used to detect morphological macrophage changes in affected mice. The upper panel of Figure 3 shows immunoreactivity in unaffected mice, and the lower panel shows the phenotype of mice with autoinflammatory disease. In the paw section, the affected mice exhibited an increase in F4/80 immunoreactivity (Figure 3F and G, arrows). F4/80 immunoreactivity also increased in the spleen in affected mice (Figure 3H, arrows). Megakaryocytes were not reacted with F4/80 and were detected in the red pulp (Figure 3H, arrowhead). Differences in macrophage immunostaining were found in the paw and spleen in eight affected mice and eight unaffected mice. In the kidney section, F4/80 immunoreactivity was found in the proximal convoluted tubules and renal cortex. We generally did not detect significant differences in F4/80 immunoreactivity between unaffected (Figure 3D and E) and autoinflammation mice in the kidney (Figure 3I and J).

### Behavioral, locomotor, and homecage tests

**Figure 4:**
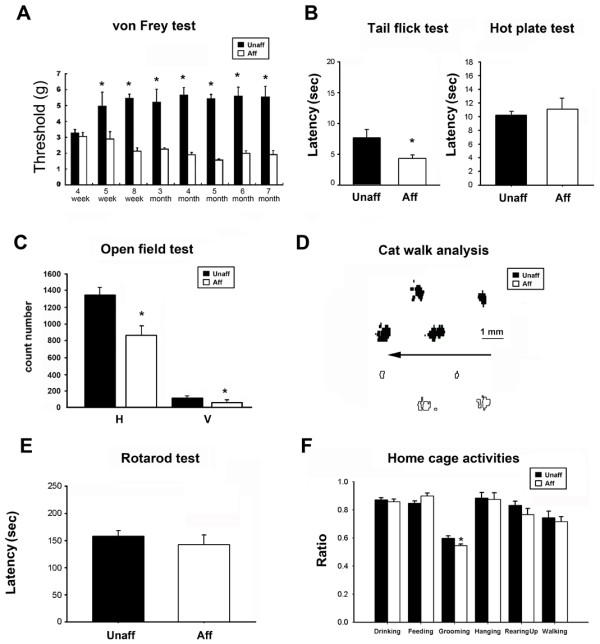
**Nociceptive and locomotor measurements.** (**A**) Nociceptive response in unaffected (*n* = 5) and affected (*n* = 8) mice from 4 weeks to 7 months. A reduction of the latency in response to mechanical stimuli was detected in affected mice. (**B**) Tail-flick and hot plate tests. The affected mice had a significantly shorter latency in the tail-flick test than unaffected mice. The latency in the hot plate test was longer in affected mice but was not significantly different from unaffected mice. (**C**) Locomotor activity in affected and unaffected mice. Locomotion in both the vertical (**V**) and horizontal (**H**) directions was lower in mice with autoinflammation than in unaffected mice. (**D**) Paw prints of affected and unaffected mice in the CatWalk analysis. The arrow indicates the gait direction. Notice that the right paw of the affected mice has significantly smaller paw prints than unaffected mice. (**E**) Rotarod test. No significant difference was found between the unaffected mice (*n* = 8) and affected mice (*n* = 10). (**F**) Homecage scanning, including drinking, feeding, hanging, rearing, grooming, and walking. A significant difference was only found in grooming between affected mice (*n* = 10) and unaffected mice (*n* = 10). Abbreviations: Unaff, unaffected mice; Aff, affected mice; V, vertical event; H, horizontal event.

The von Frey test was used to identify nociceptive behavioral responses to mechanical stimuli following the development of inflammation. The autoinflammation mice showed a reduced intensity of mechanical force to elicit signs of pain. Mechanical threshold in affected mice was significantly lower than in unaffected mice (affected mice, *n* = 8; unaffected mice, *n* = 8; *p* < 0.05; Figure 4A) from postnatal week 5 to month 7. Thus, mechanical nociceptive sensitivity was influenced by the development of an autoinflammatory syndrome. Thermal nociceptive responses were evaluated in the tail-flick and hot plate tests. A significant difference in the tail-flick response was observed between affected and unaffected mice (unaffected mice, *n* = 9; affected mice, *n* = 9; *p* < 0.05; Figure 4B). Affected mice had a lower thermal threshold than unaffected mice. Thermal threshold in the hot plate test increased in affected mice, but the difference between affected and unaffected mice was not significant (unaffected mice, *n* = 10; affected mice, *n* = 10; *p* > 0.05; Figure 4B). These results indicate that inflammatory processes may only affect mechanical nociception, demonstrated in the von Frey test.

Locomotor activity was tested in affected and unaffected mice as separate vertical and horizontal events in the open field test. Both horizontal and vertical locomotor activity was significantly reduced in autoinflammation mice (*p* < 0.01, horizontal events; *p* < 0.01, vertical events; affected mice, *n* = 11; unaffected mice, *n* = 22; Figure 4C). To test whether the changes in mechanical threshold and locomotor activity alter walking steps, the size of paw contact with the floor was calculated in wildtype and affected mice using the CatWalk protocol. Typical examples of paw prints during locomotion in affected and unaffected mice are shown in Figure 4D. The paw contact area results showed that the inflamed limb in affected mice had a smaller contact area (13.9 ± 1.9 mm^2^, *n* = 4) than the normal limb in unaffected mice (87.2 ± 8.5 mm^2^, *n* = 7, *p* < 0.01).

The rotarod test was performed, the results of which showed no significant difference between affected and unaffected mice (affected mice, *n* = 10; unaffected mice, *n* = 10; *p* > 0.05; Figure 4E). These results indicate that the reduction of mechanical threshold and locomotor activity in affected mice was not caused by motor strength weakness or loss of coordination. General health was assessed by monitoring daily activity in the homecage. Drinking, feeding, grooming, hanging, rearing, and walking were evaluated for 24 h while the mice were kept in their homecage. The results showed no significant differences in drinking, feeding, hanging, rearing, or walking. However, grooming significantly decreased (*p* < 0.05; *n* = 10; Figure 4F). These results indicate that general health was normal in affected mice compared with unaffected mice. Grooming behavior may be influenced by inflammatory conditions in the inflamed paws.

### Fos and CGRP immunostaining in the spinal cord

**Figure 5:**
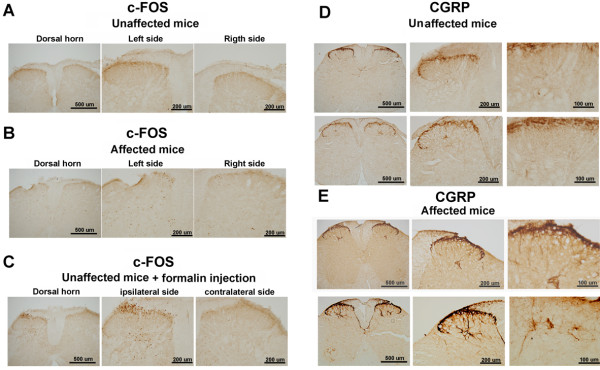
**Fos and CGRP immunoreactivity in the spinal cord in affected and unaffected mice.** (**A**) No Fos expression was found in the spinal cord in unaffected mice. (**B**) Fos immunoreactivity was detected in the dorsal horn in autoinflammation mice. (**C**) A positive control used formalin injection into the paws of unaffected mice. Fos expression was enhanced in the dorsal horn of the spinal cord. (**D**) The densest part of CGRP labeling was in the laminar II layer of the dorsal horn of the spinal cord. CGRP immunostaining revealed no detectable fibers in laminae III-V in the spinal cord in unaffected mice. (**E**) CGRP-immunoreactive fibers in laminae III-V of the spinal cord were enhanced in autoinflammation mice.

c-*fos* is a cellular proto-oncogene that forms a complex Fos protein that regulates the proliferation and differentiation of most cell types and is used to detect neuronal activity in response to noxious stimuli. We used a Fos antibody to reveal neuronal activity in the spinal cord to identify cellular changes in autoinflammation mice. The mice were sacrificed at week 8, and the spinal cords of affected and unaffected mice were removed and placed in fixative buffer. Fos immunoreactivity at low and high magnification in unaffected and affected mice is shown in Figure 5A and B, respectively. We examined Fos expression in eight affected and five unaffected mice. No significant differences in Fos expression were detected in the dorsal horn of the spinal cord in these two groups of mice (Table 1). A positive control used formalin injection into the paws of unaffected mice. Fos expression was enhanced in the dorsal horn of the spinal cord (Figure 5C). The chronic pain marker CGRP was used to detect morphological changes in a previous study that used a chronic inflammatory pain model in animals. Normal CGRP immunoreactivity was found in unaffected mice. The densest part of CGRP labeling was in the laminar II layer of the dorsal horn of the spinal cord (Figure 5D). We observed a dense distribution in five unaffected mice. In affected mice, in addition to dense CGRP labeling in lamina II, CGRP labeling was present in the fibers of laminae III-V of the spinal cord (Figure 5E). We observed a significantly increased density of CGRP in the deep layers in six affected mice (Table 1). CGRP-containing fibers appeared to be thicker in autoinflammation mice than in unaffected mice.

**Table 1:** Immunostainings of Fos, CGRP, and microglia in the central nervous system of affected and unaffected mice

	**Fos**	**CGRP**	**Microglia**		
			**Cerebral cortex**	**Hippocampus**	**Spinal cord**
Unaffected	71.65 ± 13.84 (*n* = 5)	792.33 ± 60.20 (*n* = 5)	146.10 ± 8.42 (*n* = 6)	242.83 ± 17.75 (*n* = 6)	213.15 ± 13.13 (*n* = 6)
Affected (5 weeks)	102.19 ± 13.53 (*n* = 8)	1512.23 ± 231.54* (*n* = 6)	413.85 ± 5.61* (*n* = 3)	593.12 ± 18.92* (*n* = 3)	1023.89 ± 15.04 ** (*n* = 3)
Affected (20 weeks)			500.79 ± 244.11 (*n* = 5)	361.14 ± 57.71# (*n* = 5)	247.78 ± 24.71# (*n* = 5)

Values are expressed as the density of immunostaining in a standard unit area of 200 μm × 200 μm (mean ± SEM). *p < 0.05, **p < 0.01, significant difference between the unaffected and affected (5 weeks) groups. #*p* < 0.05, significant difference between the affected (5 weeks) and affected (20 weeks) groups.

### Microglia immunoreactivity in affected and unaffected mice

**Figure 6:**
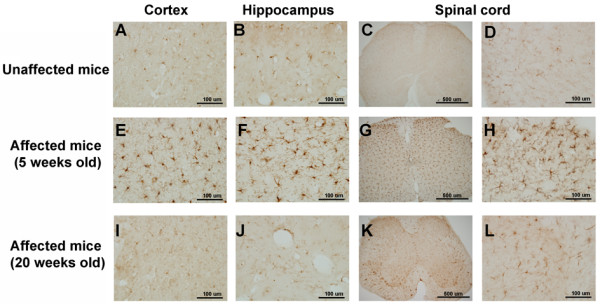
**Morphological microglial changes in unaffected and affected mice.** Microglia were stained with an Iba-1 antibody. (Upper panel) Microglia stainings in the cortex (primary somatosensory cortex), hippocampus (dentate gyrus), and spinal cord of unaffected mice. (Middle and lower panels) Microglia stainings in respective brain areas of 5-week-old and 20-week-old affected mice.

Microglia play a key role in the maintenance of chronic pain and can be activated in the neuropathic pain model. To investigate changes in microglia in the central nervous system, we used an IBa-1 antibody to detect the density of microglia in mice with autoinflammation. The microglia were stained in the spinal cord and brain slices of unaffected and affected mice. Figure 6, upper left panel, shows normal microglia in the cerebral cortex, hippocampus, and spinal cord in unaffected mice. We observed this pattern of microglia distribution in six unaffected mice. In young affected mice at 5 weeks (Figure 6, middle panel), the microglia exhibited enlargement and more fiber branching in the cerebral cortex, hippocampus, and spinal cord than in unaffected mice. We observed these phenomena in three young mice at 5 weeks. Microglia activity in five 20-week-old affected mice were reduced to normal levels in the cerebral cortex but not in the spinal cord or hippocampus compared with unaffected mice (Figure 6, right panel; Table 1).

### Identification of point mutation in autoinflammation mice

**Figure 7:**
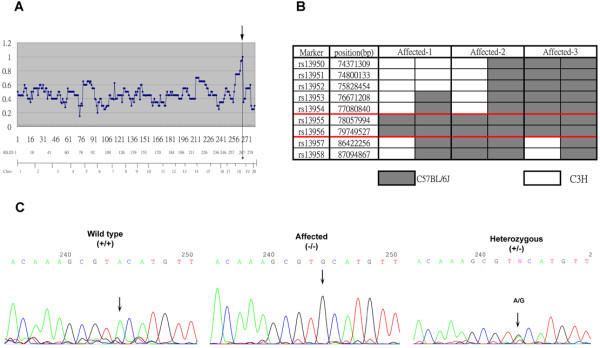
**Identification of point mutation in autoinflammation mice.** (**A**) SNP mapping of autoinflammation mutation. The figure presents the linkage of the mutant phenotype to the distal region of chromosome 18 (position 18.079.679). Two hundred eighty-five SNP markers are plotted on the x-axis, ordered by chromosome location. The graph shows the percentage of C57BL/6 J genetic background calculated from 10 autoinflammation mice. (**B**) Fine mapping of chromosome 18. The proximal border is defined by marker rs13955, whereas the distal border is defined by marker rs13956. The data were obtained from 28 affected mice. (**C**) Sequences of *Pstpip2* gene from wildtype, heterozygous, and homozygous mutant mice revealed a c.240A⋄G(Y180C) mutation in exon 8. The C57BL/6 J and C3H carry an A at the respective position. Heterozygous mice show a G/A double-peak. Autoinflammation mice carry only a G.

To identify the genetic mutation in autoinflammation mice, genome-wide SNP mapping in 42 autoinflammation mice was performed for 285 SNP markers. A representation of the linkage of the mutant phenotype is shown in Figure 7A. Strong linkage was found in the distal region of chromosome 18 (Figure 7A, arrow). The mutation was localized between markers rs13483437 (78057994 bp) and rs13483455 (86422256 bp). We then selected 10 SNP markers that narrowed the critical region to between rs13955 and rs13956 (1.7 Mb physical distance; Figure 7B). In this region, five candidate genes on the distal part of chromosome 18, including *Pstpip2*, *Nfatc1*, *Galr1*, *Mbp*, and *Cd 226* were sequenced using the mouse DNA PCR product. Sequencing all 13 exons of *pstpip2/Mayp* revealed a single A-to-G base mutation in exon 8 (Figure 7C). The identified genotypes were the following: G/G for affected homozygous mice, A/A for unaffected homozygous mice, and an A/G double-peak for unaffected heterozygous mice. The mutation was present in all affected mice (*n* = 20). According to the triplet genetic code, the mutant base was in the second position of residue 180 of *pstpip2*, resulting in a single amino acid substitution of tyrosine with cysteine at position 180 (Y180C).

## Discussion

The present study found an ENU-induced point mutation in mice that caused autoinflammatory disease. We found that the inflammatory phenotype was irreversible in the paws, skin, and ears, and this autoinflammatory disease caused an abnormal nociceptive response. Histological analysis revealed an increase in megakaryocytes in the spleen and hyperplasia of the epidermis of the paws in affected mice. Abnormal nociceptive behavior was found in affected mice, but their motor coordination and general daily activities in the homecages were normal compared with unaffected mice. Genetic mapping indicated that the autoinflammation mice showed a point mutation in *pstpip2/Mayp* located on the distal part of chromosome 18 that caused an amino acid substitution from tyrosine to cysteine.

An ENU-induced genetic point mutation in *Plcg2* and *pstpip2/Mayp* that causes autoinflammatory disease in mice has been reported [21,22]. A phospholipase Cγ2 (*Plcg2)* mutation resulted in severe spontaneous inflammation and autoimmunity. The disease caused a gain-of-function mutation in *Plcg2*, leading to hyperactive external calcium entry in B cells and an expansion of innate inflammatory cells [22]. Another study reported that a mutation in *Pstpip2* in so-called *cmo* mice resulted in bone, cartilage, and skin inflammation. This autoinflammatory phenotype was derived from L98P amino acid substitutions in *Pstpip2*[21]. Grosse and colleagues also reported an ENU-induced point mutation in macrophage actin-associated tyrosine phosphorylated protein (*Mayp/pstpip2*) [23]. This *Lupo* mouse has a T-to-A base pair change in *psptpip2/Mayp* caused by an amino acid substitution from isoleucine to asparagine (I282N), leading to autoinflammatory disease [23]. Our autoinflammation mice showed a similar phenotype as the previously reported *Lupo* and *cmo* mice. However, the mutation site (Y180C; see Figure 7) was not localized to the same exon as previous findings. This indicates that the *psptpip2/Mayp* gene has several domains, the mutation of which causes dramatic functional impairments.

Autoinflammatory diseases are described as a group of inherited disorders characterized by episodes of seemingly unprovoked inflammatory attacks of an innate origin, mainly by neutrophils [24]. An abnormal immune system causes a disruption of protection mechanisms and other complications. Autoinflammatory diseases are defined as illnesses caused by primary dysfunction of the innate immune system [25] and are systemic disorders characterized by apparently unprovoked inflammation in the absence of high titer autoantibodies or antigen-specific T-lymphocytes [26]. Six autoinflammatory diseases (i.e., pyogenic arthritis, pyoderma gangrenosum and acne [PAPA], familial Mediterranean fever (FMF), familial cold autoinflammatory syndrome [FCAS], Blau disease, tumor necrosis factor receptor-associated periodic syndrome (TRAPS), and hyper-IgD with periodic fever syndrome [HIDS]) are all inherited as a single-gene Mendelian disease [26,27]. PAPA syndrome is an autosomal-dominant disease characterized by polymorphonuclear leukocyte invasion of the joints and skin that produces destructive arthritis and skin lesions [28,29]. PAPA syndrome is correlated with a *pstpip1* mutation that causes an attenuation of the PSTPIP1 association with proline-glutamic acid-serine-threonine-rich (PEST) protein tyrosine phosphatase. The mutant protein then results in a loss of function of the innate immune response [30,31]. In a recent report, pyrin, the FMF protein, was shown to interact with *pstpip1* and CD2 binding protein 1. Mutations of *pstpip1* and CD2 binding protein 1 cause pyrin hyperphosphorylation, which modulates normal immunoregulatory function with other proteins [32]. Most patients with FMF carry missense mutations in the *C*-terminal half of the pyrin protein.

Proline, serine, and threonine phosphatase interacting protein (PSTPIP), an actin- associated protein, is involved in the assembly of the actin ring in the cytokinetic cleavage furrow. PSTPIP2 homo-oligomerizes, similar to the PSTPIP1 association with the actin cytoskeleton. Both PSTPIP1 and PSTPIP2 have a Fes/CIP4 homology domain and a putative coil-coil domain [33]. Macrophage actin-associated tyrosine-phosphorylated protein (MAYP), a protein related to PSTPIP2, regulates the CSF-1-induced reorganization of the actin cytoskeleton [34]. MAYP belongs to the Pombe Cdc15 homology (PCH) family of proteins that are involved in the regulation of actin-based functions, inhibition of actin reorganization into membrane ruffles, and stimulation of the formation of filopodia by bundling actin *in vitro* and *in vivo*. The coiled-coil region may be important for this function because it contains a putative actin-binding domain and is postulated to mediate MAYP oligomerization [35].

The analysis of the organs in autoinflammation mice revealed an epithelial layer that was thinner than in normal mice. These autoinflammatory phenotypes were irreversible in the paws (see Figure 1). Radiography revealed destruction of digital bones in affected mice. This phenomenon is similar to a previous report of *Lupo* mice. This bone destruction was also present in animals after chronic treatment with inflammatory mediators [36-38]. Research in patients also indicated that bone destruction could be induced after chronic inflammatory disease [39]. This bone erosion symptom in inflammatory patients derived from a significant increase in osteoclast precursors, leading to the generation of osteoclasts [40]. We also used MRI in affected mice and observed an enlargement of soft tissue in inflammatory areas. In a study of human autoinflammatory disease in arthritic patients, tissue edema, synovitis, bone erosion, and bone edema were reported [41]. The affected mice in the present study also exhibited infiltration by inflammatory material in the paw section, causing edema in the epidermal and dermal layers. This finding is similar to the previous reports of *cmo* and *Lupo* mice. These imaging results indicated that our autoinflammation mice possessed inflammatory characteristics. The incidence of inflammation was different between the fore- and hindlimbs, but the mechanism of this phenotype is still unclear and required further experimentation. The onset of clinical inflammation in our affected mice occurred at 5 to 8 weeks, in contrast to *cmo* mice that exhibited inflammation at 4 to 6 weeks and *Lupo* mice that exhibited inflammation at 42 ± 8 days.

Our present histological findings showed that after onset of clinic signs the autoinflammation mice exhibited an increase in megakaryocyte in the red pulp of the spleen (Figure 3C and H). The spleen, a lymphoid organ, plays a filtration role for animal blood and also stores blood and monocytes. Hegen and colleagues reported that the spleen had megakaryopoiesis inflammation in white pulp and marked extramedullary hematopoiesis in red pulp after arthritis induced in animals [42]. Zymosan-induced inflammation in mice resulted in the accumulation of megakaryocytes and plasma-like cells [43]. This evidence demonstrates that megakarocytes can be stimulated after inflammation or infection. This phenomenon of cell accumulation is similar to another report of the interaction between megakarocytes and platelets and internal pathogens and outside bacteria during different stages of inflammation [44]. A molecular study also found a downregulation of platelets and megakarocytes after genetic modification of the lipopolysaccharide (LPS)-binding Toll-like receptor 4 (TLR4) [45]. Furthermore, in rheumatoid arthritis patients, megakaryocytes regulated skeletal mass by inhibiting bone resorption while simultaneously stimulating bone formation and the regulation of osteoclastogenesis by competing with cytokines [46]. This evidence suggests that megakaryocytes play an important role in the inflammation signaling pathway. The abnormal megakaryocyte ratio found in our autoinflammation mice should be further studied to identify cellular-level changes in the spleen.

*MAYP/PSTPIP2* has been described with regard to the regulation of filopodia formation and motility in macrophages [34,35]. Our findings also present immunohistological macrophage marker F4/80 expression data that demonstrated accumulated macrophages in the inflamed paws and spleen. An inflammatory arthritis study also indicated that macrophage and dendritic cells are relevant components of the innate immune system [47,48]. A study of CFA-induced inflammation found an increased macrophage phenotype in the spleen using immunohistological and immunization methods [49]. Our present findings found an accumulation of macrophage marker F4/80 in the inflamed paws, reflecting an increased number of macrophages, and this phenotype may derive from arthritic disease. Our results are similar to reports in *Lupo* mice and other reports that discuss arthritic diseases [47].

The autoinflammatory syndrome that occurred in the mutant mice in the present study was irreversible, and this characteristic implies that this mutation could be a new chronic inflammatory pain model in mice. Fos protein is a general neuronal activity marker that can be expressed in an animal model of inflammation [50,51]. However, in the present study, the affected mice with inflammation syndrome did not exhibit altered Fos protein expression compared with unaffected mice. A previous chronic inflammation study also found that Fos activity in the dorsal horn was gradually attenuated after the acute inflammatory stage at 2–4 weeks [52]. Two days after a carrageenin injection, a decrease in Fos activation was found in the spinal cord [53], further suggesting that our affected mice were not in the acute inflammation stage. Altogether, these results may suggest that Fos protein expression in autoinflammation mice possibly returned to normal levels, indicating that they were in a stage of chronic inflammation recovery [54]. CGRP-immunoreactive fibers have been shown to increase bilaterally in arthritic rats [55]. High expression of CGRP protein has been reported to play an important role in chronic central neuropathic pain [56] and be related to the development of inflammation. In the present study, we found that our autoinflammation mice exhibited a large number of CGRP-like-immunoreactive fibers in laminae III and V of the spinal cord. Results from a pain study showed that noxious stimulus-induced hyperalgesia was not induced in CGRP-deficient mice after the induction of inflammation [57], and CGRP-like immunoreactivity could also be enhanced 21 days after CFA injection [58]. This evidence suggests that abnormal CGRP expression in the spinal cord may reflect pathophysiological mechanisms and involve the development of nociceptive responses.

Astrocytes and microglia have been recognized as key mediators of pathological and chronic pain [59]. Intrahippocampal injection of LPS and subcutaneous formalin injection in mice led to an increased number and activation of microglia in the central nervous system [60,61]. These activated microglia could release proinflammatory cytokines that cause hypersensitivity in an animal model of neuropathic pain [62]. Inhibition of microglial activation decreases neuropathy-induced hypersensitivity in behavioral tests [63,64]. We also verified microglial activation in affected mice at different ages during the initial period of inflammation (5 weeks). Microglia were activated in the spinal cord, cerebral cortex, and hippocampus. The microglial activation in the acute stage of pain was similar to a previous study of experimental animals with chemical and mechanical nerve root injury [65]. At 20 weeks, the autoinflammation mice displayed reversible microglia induction. This is also consistent with a study of microglial activation that was attributable to their protective role in the central nervous system following chronic pain [59].

The present von Frey and locomotor activity tests supported the behavior observed in autoinflammation mice. We recorded mechanical nociceptive responses from 4 weeks to 7 months and found chronic hypersensitivity responses in autoinflammation mice. Previous studies also found persistent hypersensitivity in an animal model of sciatic nerve ligation-induced chronic pain [66] and spinal cord injury model [67]. Locomotor activity testing has been suggested to be a reliable parameter for studies of chronic pain [68]. Our autoinflammation mice also exhibited a decrease in locomotor activity, indicating that the affected mice suffered from chronic inflammatory pain. The reduction of locomotor activity was not attributable to changes in motor strength or motor coordination. The rotarod test did not reveal any significant differences between unaffected and affected mice. Furthermore, homecage scanning of daily activity also showed normal behavior in affected mice, such as drinking, feeding, hanging, rearing, and walking. These results indicate that the inflammatory symptoms did not significantly interfere with daily behaviors, and these mice were able to maintain general health. Altogether, our results demonstrate another ENU-induced point mutation that causes autoinflammatory disease. This mouse strain may contribute to research in the pain field, especially in inflammatory pain studies.

## Conclusions

Our ENU-induced mutation of *pstpip2* revealed an autoinflammatory phenotype in the skin of the extremities. Additionally, these mutant mice exhibited mechanical allodynia. Pain symptoms caused by a mutation of the *pstpip* gene have been reported in autoinflammatory disease (e.g., PAPA syndrome). The clinical features of this syndrome include pyogenic arthritis, pyoderma, gangrenosum, and cystic acne [69]. Among these symptoms, pyderma gangrenosum is a very painful disease of skin [30]. Pain symptoms of PAPA syndrome have been reported in a patient after a minor traffic accident, the family members of whom were diagnosed with a mutation in the *pstpip* gene and confirmed as having PAPA syndrome [70]. Compared with most animal models of chemical-induced inflammatory pain, the present animal model of spontaneous pain has a longer duration of pain. Formalin- and carrageenan-induced pain have been recognized as short-term inflammatory pain models. CFA-induced inflammatory pain has been considered a model of long-term inflammatory pain. However, thermal and mechanical hyperalgesia induced by CFA was reduced 14 days after the injection. Spontaneous inflammatory pain in the present *pstpip2* mutant mice, measured by mechanical pain sensitivity, began in the 5th week and lasted for 7 months. The edema caused by inflammation also began at the same time and lasted for at least 22 weeks. These are the distinct features of the present model that differ from other animal models of chemical-induced inflammatory pain. Most experimental animal models of inflammatory pain induced by chemical injury do not mimic the clinical presentations of inflammatory pain in autoinflammatory disease. The pathology of inflammation-related chronic pain has been previously overlooked because of inadequate approaches to examining this issue. Thus, our *pstpip2* mutant mice provide a new model for investigating the potential mechanisms of inflammatory pain.

## Competing interests

The authors declare that they have no competing interests.

## Authors’ contributions

TCC participated in the design of the study, conducted the experiments, analyzed the data, and drafted the manuscript. JSW and WPC assisted in the nociceptive behavioral tests and data analysis. PNH and STH participated in the discussion of the experimental results and experimental design. BCS conceived the study, participated in its design and coordination, and participated in the writing of the manuscript. All of the authors discussed the results and commented on the manuscript. All authors read and approved the final manuscript.

## References

[B1] Sato J Perl ER Adrenergic excitation of cutaneous pain receptors induced by peripheral nerve injury Science 1991 251 1608 1610 10.1126/science.2011742 2011742

[B2] Tracey DJ Romm MA Yao NN Peripheral hyperalgesia in experimental neuropathy: exacerbation by neuropeptide Y Brain Res 1995 669 245 254 7712180 10.1016/0006-8993(94)01265-j

[B3] McMahon SB Cafferty WB Marchand F Immune and glial cell factors as pain mediators and modulators Exp Neurol 2005 192 444 462 10.1016/j.expneurol.2004.11.001 15755561

[B4] Singh Tahim A Santha P Nagy I Inflammatory mediators convert anandamide into a potent activator of the vanilloid type 1 transient receptor potential receptor in nociceptive primary sensory neurons Neuroscience 2005 136 539 548 10.1016/j.neuroscience.2005.08.005 16198486

[B5] Sauer SK Bove GM Averbeck B Reeh PW Rat peripheral nerve components release calcitonin gene-related peptide and prostaglandin E2 in response to noxious stimuli: evidence that nervi nervorum are nociceptors Neuroscience 1999 92 319 325 10.1016/S0306-4522(98)00731-3 10392853

[B6] Marchand F Perretti M McMahon SB Role of the immune system in chronic pain Nat Rev Neurosci 2005 6 521 532 15995723 10.1038/nrn1700

[B7] Moalem G Tracey DJ Immune and inflammatory mechanisms in neuropathic pain Brain Res Rev 2006 51 240 264 10.1016/j.brainresrev.2005.11.004 16388853

[B8] Wang LX Wang ZJ Animal and cellular models of chronic pain Adv Drug Deliv Rev 2003 55 949 965 10.1016/S0169-409X(03)00098-X 12935939

[B9] Honor P Menning PM Rogers SD Nichols ML Basbaum AI Besson JM Mantyh PW Spinal substance P receptor expression and internalization in acute, short-term, and long-term inflammatory pain states J Neurosci 1999 19 7670 7678 10460273 10.1523/JNEUROSCI.19-17-07670.1999 PMC6782496

[B10] Martindale JC Wilson AW Reeve AJ Chessell IP Headley PM Chronic secondary hypersensitivity of dorsal horn neurones following inflammation of the knee joint Pain 2007 133 79 86 10.1016/j.pain.2007.03.006 17467170

[B11] Wieseler-Frank J Maier SF Watkins LR Glial activation and pathological pain Neurochem Int 2004 45 389 395 10.1016/j.neuint.2003.09.009 15145553

[B12] Hansson E Could chronic pain and spread of pain sensation be induced and maintained by glial activation? Acta Physiol (Oxf) 2006 187 321 327 10.1111/j.1748-1716.2006.01568.x 16734769

[B13] Plasqui G The role of physical activity in rheumatoid arthritis Physiol Behav 2008 94 270 275 10.1016/j.physbeh.2007.12.012 18234247

[B14] Guyton MK Wingrave JM Yallapragada AV Wilford GG Sribnick EA Matzelle DD Tyor WR Ray SK Banik NL Upregulation of calpain correlates with increased neurodegeneration in acute experimental auto-immune encephalomyelitis J Neurosci Res 2005 81 53 61 10.1002/jnr.20470 15952172

[B15] Becher B Bechmann I Greter M Antigen presentation in autoimmunity and CNS inflammation: how T lymphocytes recognize the brain J Mol Med 2006 84 532 543 10.1007/s00109-006-0065-1 16773356

[B16] Lariviere WR Chesler EJ Mogil JS Transgenic studies of pain and analgesia: mutation or background genotype? J Pharmacol Exp Ther 2001 297 467 473 11303031

[B17] Mogil JS Grisel JE Transgenic studies of pain Pain 1998 77 107 128 10.1016/S0304-3959(98)00093-1 9766829

[B18] de Angelis MH Hrabe Flaswinkel H Fuchs H Rathkolb B Soewarto D Marschall S Heffner S Pargent W Wuensch K Jung M Genome-wide, large-scale production of mutant mice by ENU mutagenesis Nat Genet 2000 25 444 447 10.1038/78146 10932192

[B19] Nolan PM Peters J Strivens M Rogers D Hagan J Spurr N Gray IC Vizor L Brooker D Whitehill E A systematic, genome-wide, phenotype-driven mutagenesis programme for gene function studies in the mouse Nat Genet 2000 25 440 443 10.1038/78140 10932191

[B20] Chern Y Lee EH Lai HL Wang HL Lee YC Ching YH Circadian rhythm in the Ca(2+)-inhibitable adenylyl activity of the rat striatum FEBS Lett 1996 385 205 208 10.1016/0014-5793(96)00370-5 8647252

[B21] Ferguson PJ Bing X Vasef MA Ochoa LA Mahgoub A Waldschmidt TJ Tygrett LT Schlueter AJ El-Shanti H A missense mutation in pstpip2 is associated with the murine autoinflammatory disorder chronic multifocal osteomyelitis Bone 2006 38 41 47 10.1016/j.bone.2005.07.009 16122996 PMC3726202

[B22] Yu P Constien R Dear N Katan M Hanke P Bunney TD Kunder S Quintanilla-Martinez L Huffstadt U Schroder A Autoimmunity and inflammation due to a gain-of-function mutation in phospholipase C gamma 2 that specifically increases external Ca2+ entry Immunity 2005 22 451 465 10.1016/j.immuni.2005.01.018 15845450

[B23] Grosse J Chitu V Marquardt A Hanke P Schmittwolf C Zeitlmann L Schropp P Barth B Yu P Paffenholz R Mutation of mouse Mayp/Pstpip2 causes a macrophage autoinflammatory disease Blood 2006 107 3350 3358 10.1182/blood-2005-09-3556 16397132 PMC1895761

[B24] Stojanov S Kastner DL Familial autoinflammatory diseases: genetics, pathogenesis and treatment Curr Opin Rheumatol 2005 17 586 599 10.1097/bor.0000174210.78449.6b 16093838

[B25] Touitou I Notarnicola C Grandemange S Identifying mutations in autoinflammatory diseases: towards novel genetic tests and therapies? Am J Pharmacogenomics 2004 4 109 118 10.2165/00129785-200404020-00005 15059033

[B26] Galon J Aksentijevich I McDermott MF O’Shea JJ Kastner DL TNFRSF1A mutations and autoinflammatory syndromes Curr Opin Immunol 2000 12 479 486 10.1016/S0952-7915(00)00124-2 10899034

[B27] Brydges S Kastner DL The systemic autoinflammatory diseases: inborn errors of the innate immune system Curr Top Microbiol Immunol 2006 305 127 160 10.1007/3-540-29714-6_7 16724804

[B28] McDermott MF A common pathway in periodic fever syndromes Trends Immunol 2004 25 457 460 10.1016/j.it.2004.07.007 15324736

[B29] Cortis E De Benedetti F Insalaco A Cioschi S Muratori F D’Urbano LE Ugazio AG Abnormal production of tumor necrosis factor (TNF) – alpha and clinical efficacy of the TNF inhibitor etanercept in a patient with PAPA syndrome [corrected] J Pediatr 2004 145 851 855 10.1016/j.jpeds.2004.08.001 15580218

[B30] Wise CA Gillum JD Seidman CE Lindor NM Veile R Bashiardes S Lovett M Mutations in CD2BP1 disrupt binding to PTP PEST and are responsible for PAPA syndrome, an autoinflammatory disorder Hum Mol Genet 2002 11 961 969 10.1093/hmg/11.8.961 11971877

[B31] Tallon B Corkill M Peculiarities of PAPA syndrome Rheumatology (Oxford) 2006 45 1140 1143 10.1093/rheumatology/kei178 16527883

[B32] Shoham NG Centola M Mansfield E Hull KM Wood G Wise CA Kastner DL Pyrin binds the PSTPIP1/CD2BP1 protein, defining familial Mediterranean fever and PAPA syndrome as disorders in the same pathway Proc Natl Acad Sci U S A 2003 100 13501 13506 10.1073/pnas.2135380100 14595024 PMC263843

[B33] Wu Y Dowbenko D Lasky LA PSTPIP 2, a second tyrosine phosphorylated, cytoskeletal-associated protein that binds a PEST-type protein-tyrosine phosphatase J Biol Chem 1998 273 30487 30496 10.1074/jbc.273.46.30487 9804817

[B34] Yeung YG Soldera S Stanley ER A novel macrophage actin-associated protein (MAYP) is tyrosine-phosphorylated following colony stimulating factor-1 stimulation J Biol Chem 1998 273 30638 30642 10.1074/jbc.273.46.30638 9804836

[B35] Chitu V Pixley FJ Macaluso F Larson DR Condeelis J Yeung YG Stanley ER The PCH family member MAYP/PSTPIP2 directly regulates F-actin bundling and enhances filopodia formation and motility in macrophages Mol Biol Cell 2005 16 2947 2959 10.1091/mbc.E04-10-0914 15788569 PMC1142438

[B36] Ahmed AS Li J Ahmed M Hua L Yakovleva T Ossipov MH Bakalkin G Stark A Attenuation of pain and inflammation in adjuvant-induced arthritis by the proteasome inhibitor MG132 Arthritis Rheum 2010 62 2160 2169 20506183 10.1002/art.27492

[B37] Joosten LA Helsen MM Saxne T van De Loo FA Heinegard D van Den Berg WB IL-1 alpha beta blockade prevents cartilage and bone destruction in murine type II collagen-induced arthritis, whereas TNF-alpha blockade only ameliorates joint inflammation J Immunol 1999 163 5049 5055 10528210

[B38] Ippagunta SK Brand DD Luo J Boyd KL Calabrese C Stienstra R Van de Veerdonk FL Netea MG Joosten LA Lamkanfi M Inflammasome-independent role of apoptosis-associated speck-like protein containing a CARD (ASC) in T cell priming is critical for collagen-induced arthritis J Biol Chem 2010 285 12454 12462 10.1074/jbc.M109.093252 20177071 PMC2852983

[B39] Backhaus M Burmester GR Sandrock D Loreck D Hess D Scholz A Blind S Hamm B Bollow M Prospective two year follow up study comparing novel and conventional imaging procedures in patients with arthritic finger joints Ann Rheum Dis 2002 61 895 904 10.1136/ard.61.10.895 12228160 PMC1753903

[B40] Ritchlin CT Haas-Smith SA Li P Hicks DG Schwarz EM Mechanisms of TNF-alpha- and RANKL-mediated osteoclastogenesis and bone resorption in psoriatic arthritis J Clin Invest 2003 111 821 831 12639988 10.1172/JCI16069 PMC153764

[B41] McQueen FM Gao A Ostergaard M King A Shalley G Robinson E Doyle A Clark B Dalbeth N High-grade MRI bone oedema is common within the surgical field in rheumatoid arthritis patients undergoing joint replacement and is associated with osteitis in subchondral bone Ann Rheum Dis 2007 66 1581 1587 10.1136/ard.2007.070326 17491098 PMC2095325

[B42] Hegen M Keith JC Collins M Nickerson-Nutter CL Utility of animal models for identification of potential therapeutics for rheumatoid arthritis Ann Rheum Dis 2008 67 1505 1515 18055474 10.1136/ard.2007.076430

[B43] Volman TJ Hendriks T Goris RJ Zymosan-induced generalized inflammation: experimental studies into mechanisms leading to multiple organ dysfunction syndrome Shock 2005 23 291 297 10.1097/01.shk.0000155350.95435.28 15803050

[B44] Klinger MH Jelkmann W Role of blood platelets in infection and inflammation J Interferon Cytokine Res 2002 22 913 922 10.1089/10799900260286623 12396713

[B45] Andonegui G Kerfoot SM McNagny K Ebbert KV Patel KD Kubes P Platelets express functional Toll-like receptor-4 Blood 2005 106 2417 2423 10.1182/blood-2005-03-0916 15961512

[B46] Kacena MA Horowitz MC The role of megakaryocytes in skeletal homeostasis and rheumatoid arthritis Curr Opin Rheumatol 2006 18 405 410 10.1097/01.bor.0000231910.42666.31 16763462

[B47] Firestein GS Evolving concepts of rheumatoid arthritis Nature 2003 423 356 361 10.1038/nature01661 12748655

[B48] Wang J Fathman JW Lugo-Villarino G Scimone L von Andrian U Dorfman DM Glimcher LH Transcription factor T-bet regulates inflammatory arthritis through its function in dendritic cells J Clin Invest 2006 116 414 421 10.1172/JCI26631 16410834 PMC1326147

[B49] De Jesus M Nicola AM Frases S Lee IR Mieses S Casadevall A Galactoxylomannan-mediated immunological paralysis results from specific B cell depletion in the context of widespread immune system damage J Immunol 2009 183 3885 3894 10.4049/jimmunol.0900449 19684080 PMC2737596

[B50] Jergova S Cizkova D Orendacova J Cizek M Marsala J Localization of c-Fos protein in the rat spinal cord after carrageenan treatment Acta Histochem 2002 104 381 385 10.1078/0065-1281-00679 12553707

[B51] Buritova J Honore P Chapman V Besson JM Concurrent reduction of inflammation and spinal Fos-LI neurons by systemic diclofenac in the rat Neurosci Lett 1995 188 175 178 10.1016/0304-3940(95)11426-W 7609902

[B52] Abbadie C Besson JM c-fos expression in rat lumbar spinal cord during the development of adjuvant-induced arthritis Neuroscience 1992 48 985 993 10.1016/0306-4522(92)90287-C 1630632

[B53] Honore P Buritova J Besson JM Carrageenin-evoked c-Fos expression in rat lumbar spinal cord: the effects of indomethacin Eur J Pharmacol 1995 272 249 259 10.1016/0014-2999(94)00656-R 7713169

[B54] Calvino B Crepon-Bernard MO Le Bars D Parallel clinical and behavioural studies of adjuvant-induced arthritis in the rat: possible relationship with ‘chronic pain’ Behav Brain Res 1987 24 11 29 10.1016/0166-4328(87)90032-5 3580112

[B55] Kar S Gibson SJ Rees RG Jura WG Brewerton DA Polak JM Increased calcitonin gene-related peptide (CGRP), substance P, and enkephalin immunoreactivities in dorsal spinal cord and loss of CGRP-immunoreactive motoneurons in arthritic rats depend on intact peripheral nerve supply J Mol Neurosci 1991 3 7 18 10.1007/BF02896844 1715733

[B56] Bennett AD Chastain KM Hulsebosch CE Alleviation of mechanical and thermal allodynia by CGRP(8–37) in a rodent model of chronic central pain Pain 2000 86 163 175 10.1016/S0304-3959(00)00242-6 10779673

[B57] Zhang L Hoff AO Wimalawansa SJ Cote GJ Gagel RF Westlund KN Arthritic calcitonin/alpha calcitonin gene-related peptide knockout mice have reduced nociceptive hypersensitivity Pain 2001 89 265 273 10.1016/S0304-3959(00)00378-X 11166483

[B58] Calza L Pozza M Zanni M Manzini CU Manzini E Hokfelt T Peptide plasticity in primary sensory neurons and spinal cord during adjuvant-induced arthritis in the rat: an immunocytochemical and in situ hybridization study Neuroscience 1998 82 575 589 9466462 10.1016/s0306-4522(97)00272-8

[B59] Milligan ED Watkins LR Pathological and protective roles of glia in chronic pain Nat Rev Neurosci 2009 10 23 36 10.1038/nrn2533 19096368 PMC2752436

[B60] Malm TM Magga J Kuh GF Vatanen T Koistinaho M Koistinaho J Minocycline reduces engraftment and activation of bone marrow-derived cells but sustains their phagocytic activity in a mouse model of Alzheimer’s disease Glia 2008 56 1767 1779 10.1002/glia.20726 18649403

[B61] Wu Y Willcockson HH Maixner W Light AR Suramin inhibits spinal cord microglia activation and long-term hyperalgesia induced by formalin injection J Pain 2004 5 48 55 10.1016/j.jpain.2003.09.006 14975378

[B62] Ledeboer A Sloane EM Milligan ED Frank MG Mahony JH Maier SF Watkins LR Minocycline attenuates mechanical allodynia and proinflammatory cytokine expression in rat models of pain facilitation Pain 2005 115 71 83 10.1016/j.pain.2005.02.009 15836971

[B63] Raghavendra V Tanga F DeLeo JA Inhibition of microglial activation attenuates the development but not existing hypersensitivity in a rat model of neuropathy J Pharmacol Exp Ther 2003 306 624 630 10.1124/jpet.103.052407 12734393

[B64] Tsuda M Mizokoshi A Shigemoto-Mogami Y Koizumi S Inoue K Activation of p38 mitogen-activated protein kinase in spinal hyperactive microglia contributes to pain hypersensitivity following peripheral nerve injury Glia 2004 45 89 95 10.1002/glia.10308 14648549

[B65] Rothman SM Winkelstein BA Chemical and mechanical nerve root insults induce differential behavioral sensitivity and glial activation that are enhanced in combination Brain Res 2007 1181 30 43 17920051 10.1016/j.brainres.2007.08.064 PMC2174426

[B66] Seltzer Z Dubner R Shir Y A novel behavioral model of neuropathic pain disorders produced in rats by partial sciatic nerve injury Pain 1990 43 205 218 10.1016/0304-3959(90)91074-S 1982347

[B67] Hains BC Waxman SG Activated microglia contribute to the maintenance of chronic pain after spinal cord injury The Journal of neuroscience : the official journal of the Society for Neuroscience 2006 26 4308 4317 10.1523/JNEUROSCI.0003-06.2006 16624951 PMC6674010

[B68] Larsen JJ Arnt J Reduction in locomotor activity of arthritic rats as parameter for chronic pain: effect of morphine, acetylsalicylic acid and citalopram Acta Pharmacol Toxicol (Copenh) 1985 57 345 351 3867235 10.1111/j.1600-0773.1985.tb00056.x

[B69] Lachmann HJ Hawkins PN Developments in the scientific and clinical understanding of autoinflammatory disorders Arthritis Res Ther 2009 11 212 10.1186/ar2579 19232070 PMC2688228

[B70] Dierselhuis MP Frenkel J Wulffraat NM Boelens JJ Anakinra for flares of pyogenic arthritis in PAPA syndrome Rheumatology (Oxford) 2005 44 406 408 15637033 10.1093/rheumatology/keh479

